# Study on Efficient Operating Conditions for Bipolar Membrane Electrodialysis Using Different Ion Species and Anion-Exchange Membranes

**DOI:** 10.3390/membranes14120262

**Published:** 2024-12-06

**Authors:** Sadato Kikuchi, Souichiro Hirao, Shunya Kayakiri, Yuriko Kakihana, Mitsuru Higa

**Affiliations:** 1 Cement/Concrete Research Laboratory, Sumitomo Osaka Cement Co., Ltd., 585 Toyotomi-cho, Funabashi 274-8601, Japan; skikuchi@soc.co.jp; 2 Graduate School of Science and Technology for Innovation, Yamaguchi University (YU), 2-16-1 Tokiwadai, Ube 755-8611, Japan; a007fju@yamaguchi-u.ac.jp (S.H.); kakihana@yamaguchi-u.ac.jp (Y.K.); 3 Blue Energy Center for SGE Technology (BEST), YU, 2-16-1 Tokiwadai, Ube 755-8611, Japan

**Keywords:** bipolar membrane electrodialysis, current efficiency, power intensity, carbon capture utilization and storage

## Abstract

To investigate efficient operating conditions for bipolar membrane electrodialysis (BMED), a comparison of current efficiency (*CE*) and power intensity (*PI*) was conducted using different anion-exchange membranes (AEMs) and salt solutions (NaCl and Na_2_SO_4_) as feed solutions in BMED. The results indicated that *CE* was higher and *PI* was lower for a commercial proton-blocking AEM (ACM) than for a standard AEM (ASE) when NaCl was used. This is because ASE has a higher water content than ACM, leading to greater H^+^ permeability, which reduces *CE*. Conversely, when Na_2_SO_4_ was used, ASE exhibited higher *CE* and lower cell voltage (*CV*) than ACM, resulting in lower *PI* for ASE. This is attributable to the fact that, with Na_2_SO_4_, the effect of *CV* was more significant than H^+^ permeability. These findings suggest that efficient operation can be achieved by selecting the appropriate combination of AEMs and salt solutions.

## 1. Introduction

Global warming is one of the most pressing issues facing the world today. According to the IPCC’s Sixth Assessment Report (AR6), the average global temperature has risen by approximately 1.1 °C compared to pre-industrial levels, with a possibility of exceeding 1.5 °C by the 2030s [1]. Consequently, there is a strong demand for reducing greenhouse gas emissions, particularly CO_2_, a major contributor to global warming.

Annual global CO_2_ emissions from energy sources amount to approximately 37.4 billion tons, with Japan alone emitting around 1 billion tons per year [2]. Various efforts have been initiated across different industrial sectors to mitigate CO_2_ emissions.

The cement industry ranks as the fourth largest source of CO_2_ emissions in Japan, following the electricity, steel, and chemical industries. The cement manufacturing process generates significant CO_2_ emissions, primarily due to the calcination of limestone, which alone accounts for approximately 40 million tons of CO_2_ per year [3]. As a result, measures to reduce CO_2_ emissions in this sector have become increasingly urgent. One promising approach is carbon recycling (carbon capture, utilization, and storage) [4], which views CO_2_ as a resource, allowing its separation, recovery, and subsequent utilization for new purposes. Mineralization, which involves fixing CO_2_ as a mineral (e.g., an alkali carbonate), is another method being explored. Various technological advancements in this area are currently under way and are expected to be implemented in the near future.

Methods for CO_2_ separation and recovery include chemical absorption, adsorption, and membrane separation [5]. The chemical absorption method involves separating CO_2_ through chemical reactions, with an aqueous amine solution used as the CO_2_ absorption medium for efficient recovery. However, the heating process necessary to separate CO_2_ from the amine solution consumes a significant amount of energy, resulting in high costs [6]. In the adsorption method, CO_2_ is adsorbed onto an adsorbent such as pressurized activated carbon, and CO_2_ is then desorbed and recovered by reducing pressure during the desorption process. While the adsorption method demonstrates higher energy efficiency than the chemical absorption method, the adsorption and desorption cycles can be time-consuming [7]. Conversely, the membrane separation method utilizes pressure differences between gases to recover CO_2_. The primary challenges with this approach include optimizing operating conditions based on CO_2_ concentration and pressure levels, as well as enhancing the performance of the separation membrane [8].

**Figure 1:**
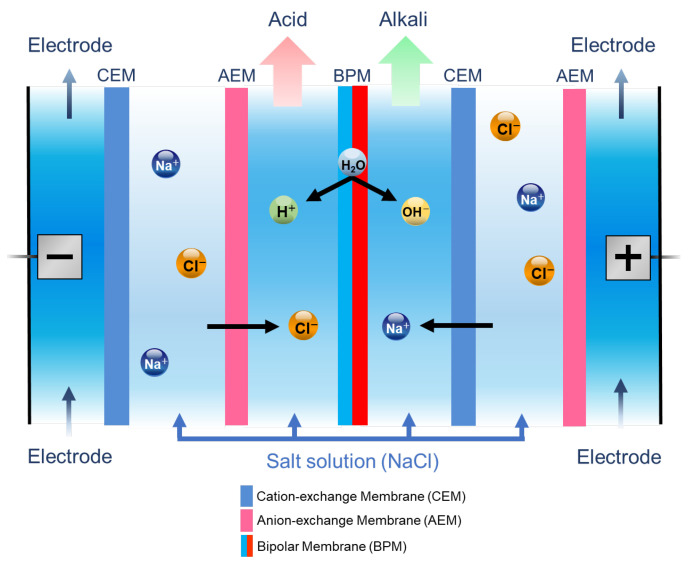
Principle of the production of acids and alkalis from salts by the three-chamber BMED.

Electrodialysis (ED) using ion-exchange membranes (IEMs) is a widely employed membrane separation method [9,10]. IEMs are categorized into cation-exchange membranes (CEMs), which are selectively permeated by cations, and anion-exchange membranes (AEMs), which are selectively permeated by anions. Bipolar membranes (BPMs) consist of both cation- and anion-exchange layers. When voltage is applied to the membrane, water at the interface between the two layers is split into hydrogen and hydroxide ions. This function allows BPMs to be used in ED to produce acids and alkalis from salts [11]. A schematic representation of the production of acids and alkalis from salts by using three-chamber bipolar membrane electrodialysis (BMED) is provided in Figure 1. In three-chamber BMED, a pair of cells consists of an AEM, an acid chamber, a BPM, an alkaline chamber, a CEM, and a salt chamber. When a negative voltage is applied to the negatively charged layer (N) of the BPM and a positive voltage to the positively charged layer (P), H^+^ and OH^−^ generated by dissociation of water at the P−N junction move to the acid chamber and alkaline chamber, respectively. Then, they react with anions (Cl^−^, etc.) that have passed through the AEM from the salt chamber and cations (Na^+^, etc.) that have passed through the CEM from the salt chamber to simultaneously generate acid and alkali. BMED is utilized in various fields, including the recovery of lithium hydroxide from lithium salt lakes and the treatment of waste liquids [12,13,14].

**Figure 2:**
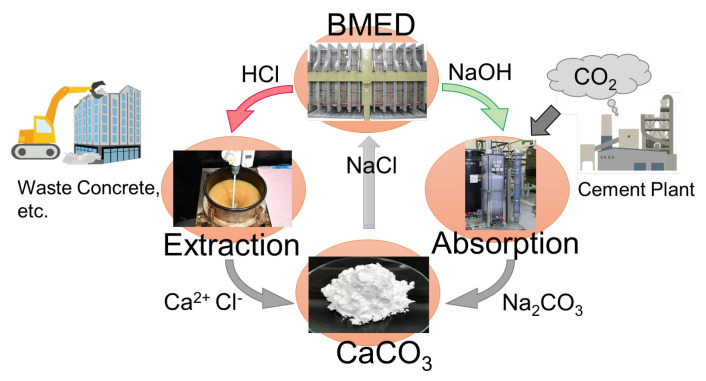
Mineralization process with BMED.

In Japan, innovative technology development is currently under way in various fields aimed at achieving carbon neutrality by 2050 [15]. This study proposes a process that reduces CO_2_ emissions by directly utilizing the CO_2_ in exhaust gases emitted from cement factories, eliminating the need for CO_2_ separation and recovery. A schematic of this process is shown in Figure 2 [16,17]. This method involves generating acids and alkalis from salt water using BMED, extracting calcium from calcium-containing waste with an acid solution, absorbing CO_2_ into the alkali solution to produce a sodium carbonate solution, and subsequently fixing CO_2_ as calcium carbonate by reacting these substances together. The efficiency of acid and alkali production using BMED is critical to this process [18].

One of the challenges of BMED is its low energy efficiency. A contributing factor to this low energy efficiency is the permeation of H^+^ ions from the AEM. Specifically, the H^+^ generated in the BPM permeates through the AEM [19], resulting in a decrease in acid production and, consequently, a decrease in energy efficiency. However, few studies have quantitatively evaluated H^+^ permeation from AEMs in the context of BMED. Additionally, there is a lack of studies that have evaluated and compared the operating conditions of different ion and membrane species used in BMED.

Therefore, this study conducted ED experiments using small cells to calculate the permeation flux of H^+^ through the AEMs and to quantitatively evaluate H^+^ leakage. In the BMED setup, the concentration in each ED cell and the cell voltage (*CV*) applied to the IEMs were measured using different AEMs and various salt solutions fed into the system. The current efficiency (*CE*) and power intensity (*PI*) of acid and alkali production were calculated to determine the optimal operating conditions.

## 2. Materials and Methods

### 2.1. Materials

The following membranes were used: a standard CEM (Neosepta® CSE), a BPM (Neosepta® BPU), a standard AEM (Neosepta® ASE), and a proton-blocking AEM (Neosepta® ACM). All the membranes were purchased from Astom Corp., Tokyo, Japan. Hydrochloric acid (HCl), sodium chloride (NaCl), and sulfuric acid (H_2_SO_4_) were obtained from NACALAI TESQUE, Inc., Kyoto, Japan. Sodium sulfate (Na_2_SO_4_) and sodium hydroxide (NaOH) were obtained from FUJIFILM Wako Pure Chemical Corp., Osaka, Japan. 

### 2.2. Characterization of Ion-Exchange Membrane

The evaluated parameters included the dynamic state transport number (*t*), membrane resistance (*R*), water content (*W*), and ion-exchange capacity (*IEC*). The characterization was performed using the same methods and instruments reported elsewhere [20,21]. 

### 2.3. Measurement of Membrane Water Content

To measure the membrane water content *W*, the membrane was removed from the 0.1 mol/L NaCl solution and dabbed with filter paper to remove excess water from its surfaces, and the weight of the wet sample membrane immersed in NaCl solution (the counter-ion of the membrane was Na^+^) *W_w_* was measured. The value of the weight in a dry state *W_d_* measured at the ion-exchange capacity (*IEC*) was used [21]. The volumetric water content was calculated from *W_w_* and *W_d_* as follows:

(1) $$W=\frac{W_{w}-W_{d}}{W_{d}}$$

### 2.4. Measurement of Ion-Exchange Capacity (IEC)

*IEC* is defined as the milliequivalent of cation-exchange groups per 1 g of dry membrane whose counter-ions are Na^+^ ions (meq/g-dry-Na form). To measure the *IEC* of a sample membrane, the membrane of 5 cm×5 cm was immersed in 0.1 mol/L KCl solution for 24 h to exchange the counter-ions for K^+^ ions. The membrane was then immersed in 0.5 mol/L of NaNO_3_ solution with a volume of 50 cm^3^ under stirring for 24 h to ensure that all of the K^+^ counter-ions were exchanged with the Na^+^ ions in the solution. The concentration of K^+^ ions *C_K_*^+^ in the solution was measured using an ion chromatograph (Dionex Integrion RFIC System, Thermo Fisher Scientific, Sunnyvale, CA, USA). The membrane was dried under vacuum for 24 h, and its dry weight *W_d_* was measured [21]. The *IEC* of the membrane was obtained using the following equation:

(2) $$\mathrm{IEC}=\frac{C_{K+}}{W_{d}}\times \frac{100}{1000}$$

For an AEM, IEC is defined as the milliequivalent of anion-exchange groups per 1 g of dry membrane whose counter-ions are Cl^−^ ions (meq/g-dry-Cl form), measured in the same way as CEM.

### 2.5. Measurement of Membrane Resistance

The electrical resistances of the membranes immersed in 0.5 mol/L NaCl were measured using an acrylic plastic cell with an effective measurement area of 1.0 cm^2^. During the measurement, 10 kHz AC (A&D Company, Ltd., Tokyo, Japan) was applied to Pt electrodes inside the cell. First, the electrical resistance Ro of the 0.5 mol/L NaCl solution was measured at 25±0.5 °C. Subsequently, a sample membrane was set in the cell, and the resistance R_s_ was measured. The membrane resistance R_m_ was calculated by subtracting R_o_ from R_s_ [20].

### 2.6. Measurement of Dynamic State Transport Number

The dynamic state transport number *t* of a sample membrane was determined via electrodialysis (ED) performed using a cell with two chambers separated by a membrane. Direct current was applied between the two electrodes (Ag–AgCl) of the cell containing 0.5 mol/L NaCl in the two chambers at a current density of 10 mA/cm^2^ at 25 °C. The conductivity change across the two chambers during ED was measured in order to obtain the equivalent change due to ion transport through the membrane [20]. The dynamic state transport number was obtained in terms of the equation:

(3) $$t=\frac{∆mVF}{Q}$$

where Δ*m*, *V*, and *Q* are the equivalent change of ions, volume of the solution, and electric charge passing through the membrane during the ED test, respectively.

### 2.7. Measurement Condition of Small-Cell ED Test

**Figure 3:**
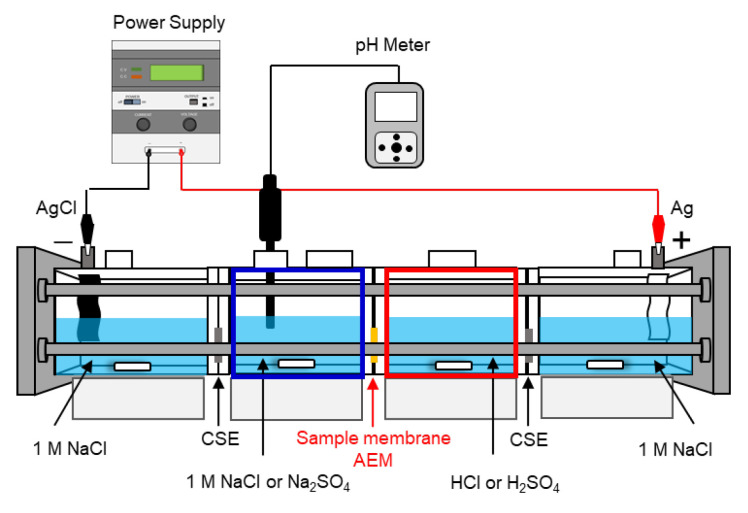
Small-cell electrodialysis (ED) experimental setup diagram.

To quantitatively evaluate the amount of H^+^ permeation from the AEMs, an ED test was conducted using a small cell, as illustrated in Figure 3. The cell consists of four compartments: two for electrodes, one for salt, and one for acid. A CEM (CSE) was placed between the electrode and salt compartments and between the electrode and acid compartment, respectively, while the acid, salt, and electrode compartments were designed to prevent solution mixing. We used Ag and AgCl electrodes (KENIS Ltd., Osaka, Japan) as the anode and cathode, respectively. The test conditions are detailed in Table 1. Sample membranes (ASE, ACM) with an effective membrane area of 4 cm^2^ were positioned between the salt and acid chambers, and ED tests were conducted using 1 mol/L NaCl or Na_2_SO_4_ as the salt solution at a current density of 25 mA/cm^2^ for 30 min. Each chamber was stirred at 2500 rpm using a stirring blade to ensure uniformity of the solution concentration in the chambers. The pH was measured using a pH meter (LAQUA WQ-320J), pH sensor head (300−P−2), and pH sensor cartridge (300−P−C). All the pH instruments were purchased from HORIBA Ltd., Kyoto, Japan. The H^+^ concentration was determined from the pH change in the salt chamber, and the permeation flux of H^+^ (*J*_i_) was calculated from the slope of the time–concentration curve of H^+^ during the ED test, $\frac{∆C_{i}}{∆t},$ using Equation (4), where *S* and *V* are the membrane area and chamber volume, respectively.

(4) $$J_{i}=\frac{V{∆C}_{i}}{S∆t}$$

### 2.8. BMED Test

**Figure 4:**
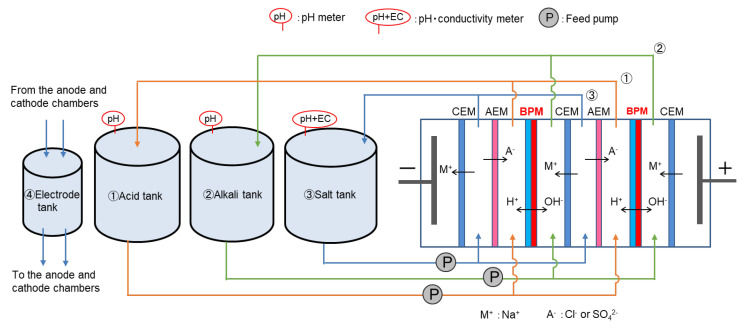
BMED experimental setup diagram. Although only two cells are shown in this diagram, there are actually 10 cells between the electrodes.

The BMED test was performed using a BMED system (EX-3B; ASTOM Corp. Tokyo, Japan) shown in Figure 4. The system stack comprised ten pairs of unit cells situated between two electrode chambers. The BMED tests were performed using nickel plates (ASTOM Corp. Tokyo, Japan) as electrodes in the electrode chambers under constant current conditions at 25 °C and 4.4 A. The total effective membrane area and chamber thickness were 550 cm^2^ (55 cm^2^ × 10) and 750 μm, respectively. The electrode, acid, alkaline, and salt chambers were connected to respective solution tanks, and each solution was fed into the chambers using a feed pump. The solution conditions are detailed in Table 2. The initial volume of each solution was 500 mL. Before the first use, the membrane was pretreated as follows: The membranes were set in the stack of the device, and 500 mL of 0.01 mol/L HCl was put in the acid and sample solution tanks; 500 mL of 0.01 mol/L NaCl was put in the alkaline and electrode solution tanks. The solutions were then pumped for 1 h to circulate the solutions. At the start of the operation, 1 mol/L NaCl or Na_2_SO_4_ was used as the salt solution, 0.001 mol/L NaCl or Na_2_SO_4_ was used as the acid and alkaline solution, and 1 mol/L NaOH was used as the electrode solution. The concentrations of the acidic, alkaline, and salt solutions were measured based on the conductivity of each solution. The volume change of each solution was determined by measuring its weight using an electronic balance (FX-3000i WP, A&D Company, Ltd., Tokyo, Japan). Using these values, the *CE* was calculated using Equation (5), where Δ*m* represents the mole equivalents of acid and alkali production during the test, while F is the Faraday constant. *N*, *I*, and ∆t are the number of pairs, electric current, and time during which the current was applied during the test.

(5) $$\mathrm{CE}E=\frac{∆m\times F}{N\times I\times t}\times 100$$

The *PI* was calculated using Equation (6). Here, *E* (kWh) is the electric energy, and *M* (kg) is the amount of acid or alkali produced. Consequently, the *PI* can be expressed in terms of the *CV* (*V*cell: V); *CE* (%); *M*_w_ (weight per mole: kg/mol); and a constant α, which is 0.67 for the production of NaOH, 0.74 for the production of HCl, and 0.27 for the production of Na_2_SO_4_.

(6) PI=E kWhMkg=CV V×I c/s×3600 s×0.001Ics÷Fcmol×3600(s)×CE×Mw (kg/mol)=CV CE×α

## 3. Results

### 3.1. Characteristics of the IEMs

The characteristics of commercially available IEMs used in this study are shown in Table 3. Although the ACM exhibited a slightly lower transport number, all membranes demonstrated high transport numbers exceeding 0.94, indicating that these membranes exhibit high counter-ion selectivity when a 0.5 M NaCl solution was used. The membrane resistance was lowest for the CSE, followed by the ASE, with the highest resistance observed in the ACM. This difference is partly attributable to the membrane water content, which was highest for the CSE, followed by the ASE, and lowest for the ACM.

### 3.2. Small-Cell ED Test

In acid and alkali production using BMED, the leakage of H⁺ ions through the AEM is one factor that reduces *CE*. To investigate the ion transport behavior of the AEM, the proton permeabilities of the AEMs were evaluated in a small-cell ED setup (Figure 3) using HCl and H_2_SO_4_.

**Figure 5:**
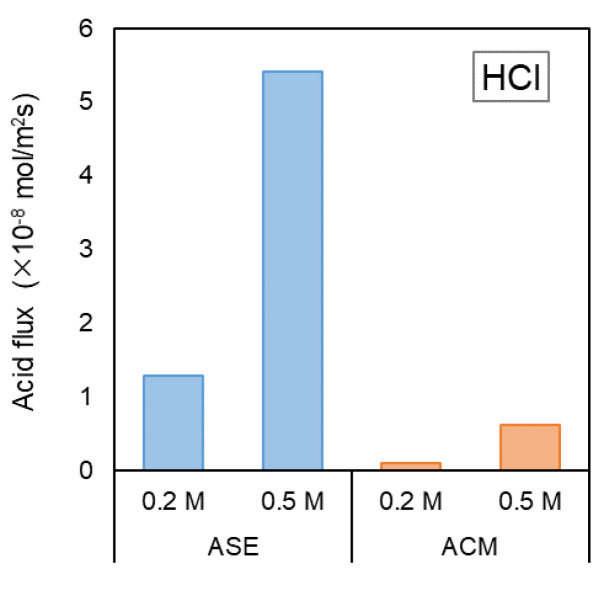
Relationship between acid flux and HCl concentration in the small-cell ED test.

**Figure 6:**
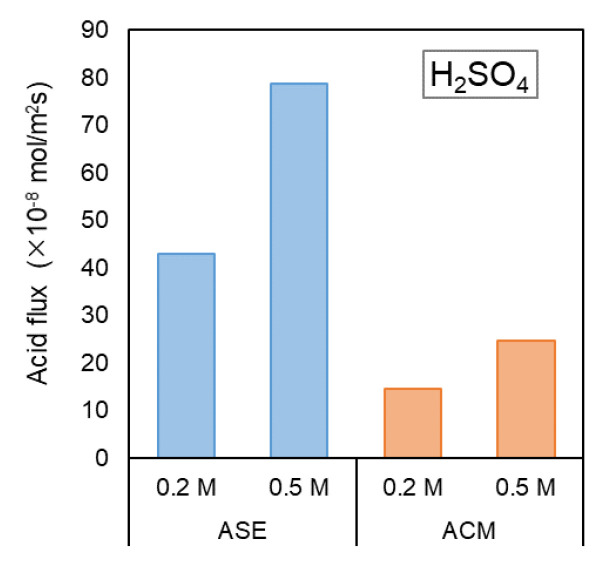
Relationship between acid flux and H_2_SO_4_ concentration in the small-cell ED test.

Figure 5 illustrates the relationship between the concentration of HCl in the acid chamber and the permeation flux of H^+^ from each AEM when NaCl and HCl were used in the salt and acid solution chambers, respectively. Additionally, Figure 6 presents the relationship between the concentration of H_2_SO_4_ in the acid chamber and the permeation flux of H^+^ from each membrane when Na_2_SO_4_ and H_2_SO_4_ were used.

**Figure 7:**
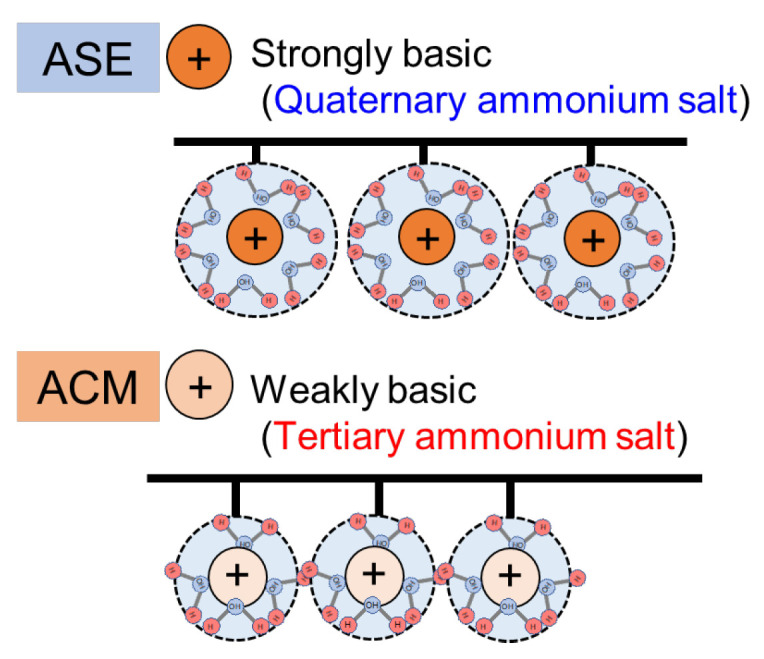
Differences in positively charged structure between ASE and ACM.

In both hydrochloric acid and sulfuric acid solutions, the permeation flux of H^+^ tended to increase as the acid concentration increased. The H^+^ flux through the ASE was greater than that through the ACM, indicating that the ASE exhibited higher H^+^ permeability than the proton-blocking AEM, ACM. In the case of HCl, the H^+^ flux through ASE was 1.30 × 10^−8^ mol/m^2^s at an acid concentration of 0.2 mol/L, whereas that through ACM was 0.01 × 10^−8^ mol/m^2^s, showing that the ACM had approximately 1/130 of the H^+^ flux of the ASE. At 0.5 mol/L, the H^+^ flux through in ASE was 5.42 × 10^−8^ mol/m^2^s, whereas that in ACM was 0.62 × 10^−8^ mol/m^2^s, indicating that the ACM exhibited 1/10 of the H^+^ permeability than ASE. In the case of H_2_SO_4_, the H^+^ flux through the ASE was 50.0 × 10^−8^ mol/m^2^s at 0.2 mol/L, whereas the H^+^ flux in ACM was 14.6 × 10^−8^ mol/m^2^s, showing that the ACM had approximately 1/3 of the H^+^ permeability of the ASE. At 0.5 mol/L, the H^+^ flux through the ASE was 78.6 × 10^−8^ mol/m^2^s, whereas that through the ACM was 24.7 × 10^−8^ mol/m^2^s, confirming that the ACM had 1/3 of the H^+^ permeability. Figure 7 illustrates the difference in the anion-exchange groups between ASE and ACM. ASE is strongly basic, with a quaternary ammonium salt as the anion charge group, while ACM is weakly basic, featuring a tertiary ammonium salt [19]. The strong basicity of ASE leads to a higher water content in the membrane, resulting in greater H^+^ permeation [22]. 

Another significant finding is that the ACM demonstrated better proton-blocking properties when the anion was chloride than when the anion was sulfide. The H^+^ permeability of H_2_SO_4_ is significantly higher than that of HCl, and at an acid concentration of 0.5 mol/L, the permeability of H_2_SO_4_ is more than 40 times higher than that of HCl. This difference can be attributed to the ion mobility of Cl^−^ and SO_4_^2−^ in water. The ion mobility of Cl^−^ in water is 8.20 × 10^−13^ molm^2^/Js, whereas that of SO_4_^2−^ is 4.30 × 10^−13^ molm^2^/Js, making the mobility of Cl^−^ approximately twice that of SO_4_^2−^ [23]. In an ED system consisting of an AEM and an acid solution, the current comprises the movement of H^+^ to the cathode and the movement of anions to the anode. Therefore, it is posited that a solution containing anions such as SO_4_^2−^ ions, which have lower mobility in the membrane, will exhibit a greater amount of H^+^ leakage than a solution containing anions such as Cl^−^ ions, which have higher mobility in the membrane. Hence, H^+^ leakage becomes less dependent on the structural differences in the AEM when using Na_2_SO_4_ as opposed to Cl^−^. 

### 3.3. BMED Test with NaCl

**Figure 8:**
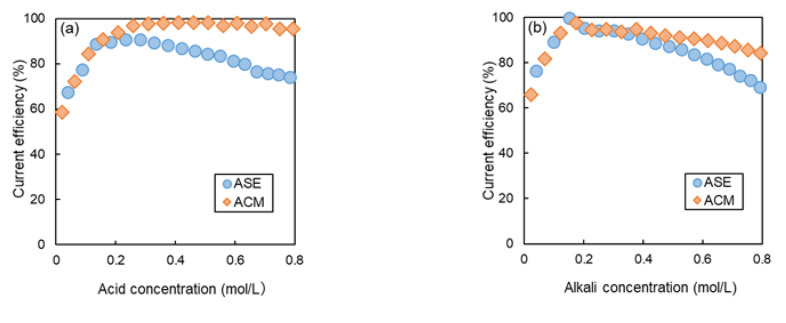

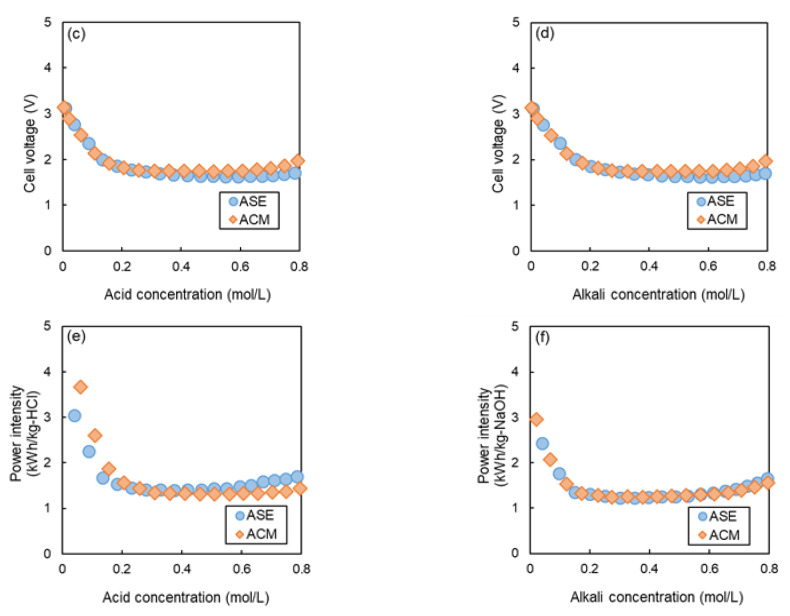
Relationship between BMED performances and acid and alkali production concentration in the case of NaCl as a feed salt solution. (**a**) *CE* at different acid concentrations, (**b**) *CE* at different alkali concentrations, (**c**) *CV* at different acid concentrations, (**d**) *CV* at different alkali concentrations, (**e**) *PI* at different acid concentrations, and (**f**) *PI* at different alkali concentrations.

Figure 8a,b illustrate the relationship between the *CE* and the concentrations of acid and alkali production when ASE and ACM were used as the AEMs, respectively. At an acid concentration of 0.5 mol/L, the *CE* of ASE was 85.7%, while that of ACM was 98.1%. When the alkali concentration was also at 0.5 mol/L, the efficiency was 87.4% for the ASE and 92.0% for the ACM. A slight decrease in *CE* was observed with increasing concentration for ASE, while the decrease in *CE* was mitigated in ACM. In BMED, as the concentrations of the generated acid and alkali increase, the concentration of the salt solution decreases, leading to a greater concentration difference between the chambers. This results in increased leakage of acid and alkali, which causes a decrease in *CE* during acid and alkali production. In this study, the *CE* of ACM was greater than that of ASE at all concentrations. Since ACM has lower H^+^ permeability than ASE, as shown in Figure 5, the *CE* of ACM is consequently higher than that of ASE. Additionally, when comparing the *CE* for acid and alkali, the *CE* for alkali was found to be higher than that for acid. This difference can be attributed to the variation in ion mobility in water between H^+^ and OH^−^.

Figure 8c,d show the relationship between acid concentration and *CV*. In both the relationship between the acid and alkali concentration, the *CV* of ACM was slightly higher than that of ASE at all tested concentrations, which can be attributed to the higher membrane resistance of ACM compared to ASE. 

Figure 8e,f present the relationship between the concentrations of acid and alkali production and the *PI* calculated using Equation (6). Despite ACM having a higher *CV* than ASE due to its greater membrane resistance, the *PI* of ACM was lower than that of ASE at concentrations about 0.2 M or more, owing to the higher *CE* of ACM. 

Table 4 lists the *CV*, *CE*, and *PI* values at acid and alkali concentrations of 0.2 mol/L and 0.5 mol/L. At an acid concentration of 0.2 mol/L, the *PI* was 1.54 kWh/kg−HCl for ASE, while it was 1.57 kWh/kg−HCl for ACM, indicating that ACM exhibited approximately 2% higher *PI* than ASE. For the 0.5 mol/L concentration, the *PI* for ASE was 1.42 kWh/kg−HCl, whereas that of ACM was 1.31 kWh/kg−HCl, showing that the *PI* of ACM was about 8% lower than ASE. At an alkali concentration of 0.2 mol/L, the *PI* for ASE was 1.31 kWh/kg−NaOH, while that of ACM was 1.32 kWh/kg−NaOH, reflecting that ACM had about 1% higher *PI* than ASE. For the 0.5 mol/L concentration, the *PI* for ASE was 1.26 kWh/kg−NaOH, while that of ACM was 1.27 kWh/kg−NaOH, with ACM showing approximately 1% higher *PI* than ASE. These results suggest that when the salt solution in BMED is NaCl, ACM can generate acids and alkalis in BMED with a higher *CE* than ASE.

### 3.4. BMED Test Using Na_2_SO_4_ as Feed Salt Solution

**Figure 9:**
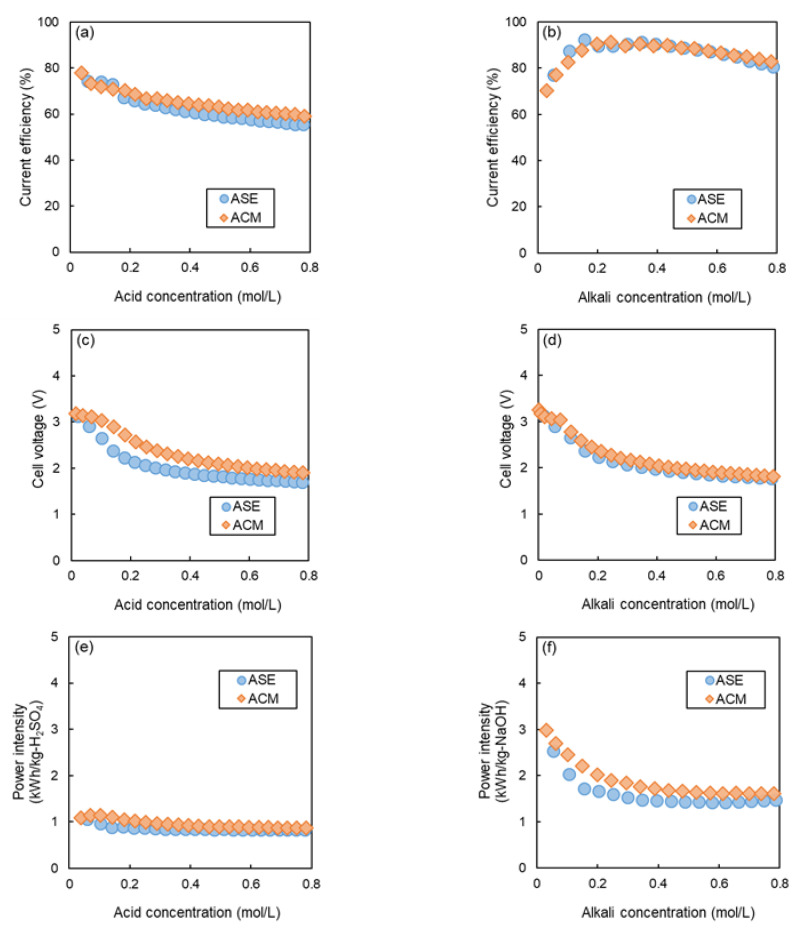
Relationship between BMED performances and acid and alkali production concentrations in the case of Na_2_SO_4_ as a feed salt solution. (**a**) *CE* at different acid concentrations, (**b**) *CE* at different alkali concentrations, (**c**) *CV* at different acid concentrations, (**d**) *CV* at different alkali concentrations, (**e**) *PI* at different acid concentrations, and (**f**) *PI* at different alkali concentrations.

Figure 9a,b illustrate the relationship between the concentrations of acid and alkali production and the *CE* when Na_2_SO_4_ was used as the salt solution.

In alkaline production, the *CE* of ASE was higher than that of ACM at all concentrations, although the difference was small, indicating that the trend was opposite to that observed with NaCl. On the other hand, in acid production, ACM showed slightly higher *CE* than ASE at concentrations of about 0.2 M or more, indicating a similar trend to that observed with NaCl.

This suggests that efficient operation can be achieved by selecting the most suitable membrane type based on the salt solution used. In the case of NaCl, almost no decrease in *CE* was observed, even at high concentrations; however, for Na_2_SO_4_, a decrease in CE was evident in both AEMs. This discrepancy can be attributed to the difference in ion mobility between Cl^−^ and SO_4_^2−^ in the solution, as discussed in Section 3.2. 

Figure 9c,d presents the relationship between the concentration of the acid produced and *CV*. At all tested concentrations, the *CV* of ACM was higher than that of ASE. At acid and alkali concentrations of 0.2 mol/L, the *CV* was 2.72 V for ACM and 2.23 V for ASE, indicating that ASE exhibited approximately 18% lower *CV* than ACM. At 0.5 mol/L, the *CV* for ASE was 1.91 V, while that for ACM was 2.20 V, resulting in ASE having about 13% lower *CV* than ACM. 

Figure 9e,f depict the *PI* as a function of acid and alkali production, respectively. Contrary to the case of NaCl, at all concentrations, the *PI* of ASE was lower than that of ACM, indicating that in the case of Na_2_SO_4_, the contribution of *CV* to the *PI* was greater than that of *CE*. 

Table 5 summarizes the *CV*, *CE*, and *PI* values at acid and alkali concentrations of 0.2 mol/L and 0.5 mol/L. At an acid concentration of 0.2 mol/L, the *PI* was 0.90 kWh/kg−H_2_SO_4_ for ASE and 1.04 kWh/kg−H_2_SO_4_ for ACM, indicating that ASE had approximately 14% lower *PI* than ACM. At 0.5 mol/L, the *PI* of ASE was 0.84 kWh/kg−H_2_SO_4_, whereas that for ACM was 0.92 kWh/kg−H_2_SO_4_, resulting in ASE being about 9% lower in *PI* than ACM. At an alkali concentration of 0.2 mol/L, the *PI* was 1.67 kWh/kg−NaOH for ASE and 2.02 kWh/kg−NaOH for ACM, reflecting that ASE was about 17% lower in *PI* than ACM. For 0.5 mol/L, the *PI* was 1.44 kWh/kg−NaOH for ASE and 1.66 kWh/kg−NaOH for ACM, which is approximately 13% lower than that of ACM. These results indicate that ASE generates acids and alkalis in BMED with lower energy efficiency than ACM in the case of Na_2_SO_4_. While ACM exhibits low H^+^ permeability due to its low water content, it simultaneously has low anion permeability. Specifically, the mobility of SO_4_^2−^ ions significantly decreases in the membrane due to their larger hydration radius, which enhances proton permeability. Consequently, for SO_4_^2−^ ion, the difference in H^+^ permeability between ACM and ASE diminishes, suggesting that the power consumption rate of ACM becomes higher than that of ASE due to the influence of ACM’s higher *CV*.

## 4. Conclusions

One of the challenges in improving the operating efficiency of BMED is reducing the permeation of H^+^ ions from the AEM in BMED. In this study, the permeation flux of H^+^ between the commercial standard AEM, ASE, and the commercial proton-blocking AEM, ACM, was evaluated using different acidic solutions via the small-cell ED test to quantitatively evaluate H^+^ leakage. The results indicated that under both HCl and H_2_SO_4_ conditions, the H^+^ permeation flux increased with higher acid concentrations, with ASE exhibiting greater H^+^ permeation than ACM. This can be attributed to ASE being strongly basic because it has a quaternary ammonium salt as the anion charge group, while ACM is weakly basic, utilizing a tertiary ammonium salt. The strong basicity of ASE leads to higher water content, which facilitates greater H⁺ permeation compared to ACM. 

Furthermore, we compared the effects of HCl and H_2_SO_4_ in salt solutions. The chloride ion demonstrates higher mobility in water; consequently, in an ED system, a solution containing anions with low mobility in the AEM, such as SO_4_^2−^ ions, results in greater H^+^ leakage compared to solutions with high-mobility anions such as Cl^−^ ions.

The BMED tests revealed that when NaCl was used as the salt solution, ACM exhibited higher *CE* and lower *PI* than ASE. Conversely, when Na_2_SO_4_ was employed as the salt solution, ASE demonstrated higher *CE* and lower *PI* than ACM. This indicates that in the case of NaCl, minimal H^+^ leakage occurs due to the high mobility of Cl^−^ in the AEM. In contrast, Na_2_SO_4_ resulted in significant H^+^ leakage due to the low mobility of SO_4_^2−^ in the AEM. Therefore, H^+^ leakage becomes less dependent on the structural differences in the AEM when using Na_2_SO_4_ as opposed to Cl^−^. As a result, ASE with a lower cell voltage showed a lower *PI* than ACM.

These results clearly demonstrate that efficient BMED operating conditions can be achieved through an optimal combination of membrane properties and the types of salt solutions utilized in the process.

## Figures and Tables

**Table 1:** Small-cell ED experimental conditions.

Salt Compartment		Acid Compartment	
Solution	Conc. (mol/L)	Solution	Conc. (mol/L)
NaCl	1.0	HCl	0.2
			0.5
Na_2_SO_4_	1.0	H_2_SO_4_	0.2
			0.5

**Table 2:** Solutions used in BMED tests.

AEM	Electrode Compartment		Salt Compartment		Acid Compartment		Alkali Compartment	
	Solution	Conc. (mol/L)	Solution	Conc. (mol/L)	Solution	Conc. (mol/L)	Solution	Conc. (mol/L)
ASE	NaOH	1.0	NaCl	1.0	NaCl	0.001	NaCl	0.001
ACM								
ASE	NaOH	1.0	Na_2_SO_4_	1.0	Na_2_SO_4_	0.001	Na_2_SO_4_	0.001
ACM								

**Table 3:** Characteristics of the IEMs used in this study: *t*, transport number; *R*, membrane resistance; *W*, water content; *IEC*, ion-exchange capacity; *d*, membrane thickness.

		*t* (−)	*R* (Ωcm^2^)	*W* (%)	*IEC* (meq/g)	*d* (µm)
CEM	CSE	0.99	2.03	27.0	1.98	148
AEM	ASE	0.99	2.77	16.0	1.96	150
	ACM	0.94	3.23	15.0	1.45	110

**Table 4:** *CV*, *CE*, and *PI* at each concentration of acid and alkali in BMED.

AEM	Salt Compartment		Acid Compartment				Alkali Compartment					
	Solution	Conc. (mol/L)	Solution	Conc. (mol/L)	Current Efficiency (%−HCl)	Power Intensity (kWh/kg−HCl)		Solution	Conc. (mol/L)	Current Efficiency (%−NaOH)	Power Intensity (kWh/kg−NaOH)	Cell Voltage (V)
ASE	NaCl	1.0	HCl	0.2	89.7	1.54		NaOH	0.2	95.5	1.31	1.86
				0.5	85.7	1.42			0.5	87.4	1.26	1.64
ACM	NaCl	1.0	HCl	0.2	90.6	1.57		NaOH	0.2	97.6	1.32	1.92
				0.5	98.1	1.31			0.5	92.0	1.27	1.74

**Table 5:** *CV*, *CE*, and PI at each concentration of acid and alkali in BMED.

AEM	Salt Compartment		Acid Compartment				Alkali Compartment				
	Solution	Conc. (mol/L)	Solution	Conc. (mol/L)	Current Efficiency (%−H_2_SO_4_)	Power Intensity (kWh/kg−H_2_SO_4_)	Solution	Conc. (mol/L)	Current Efficiency (%−NaOH)	Power Intensity (kWh/kg−NaOH)	Cell Voltage(V)
ASE	Na_2_SO_4_	1.0	H_2_SO_4_	0.2	67.3	0.90	NaOH	0.2	89.6	1.67	2.23
				0.5	61.2	0.84		0.5	88.9	1.44	1.91
ACM	Na_2_SO_4_	1.0	H_2_SO_4_	0.2	70.3	1.04	NaOH	0.2	90.4	2.02	2.72
				0.5	64.6	0.92		0.5	88.9	1.66	2.20

## Data Availability

The original contributions presented in the study are included in the article, further inquiries can be directed to the corresponding author.
